# The efficacy, pharmacokinetics, safety and cardiovascular risks of switching nevirapine to rilpivirine in HIV-1 patients: the RPV switch study

**DOI:** 10.7448/IAS.17.4.19789

**Published:** 2014-11-02

**Authors:** Casper Rokx, Maren Blonk, Annelies Verbon, David Burger, Bart JA Rijnders

**Affiliations:** 1 Department of Internal Medicine, Erasmus University Medical Center, Rotterdam, Netherlands; 2 Department of Pharmacology, Radboud University Medical Center, Nijmegen, Netherlands

## Abstract

**Introduction:**

Nevirapine (NVP) induces cytochrome P450 3A4 by which rilpivirine (RPV) is metabolized. Switching NVP to RPV could result in decreased RPV exposure with subsequent virological failure and dyslipidemia because NVP is regarded as the least dyslipidemic, non-nucleoside, reverse transcriptase inhibitor. This trial evaluated the efficacy, pharmacokinetics, safety and cardiovascular risks of switching NVP to RPV.

**Materials and Methods:**

Prospective open label controlled trial. HIV-1 patients with HIV-1 RNA <50 copies/mL on once daily NVP, emtricitabine/tenofovir (FTC/TDF) switched to single tablet RPV/FTC/TDF. Eligible patients on NVP, FTC/TDF were controls. Primary endpoint was week 12 HIV-1 RNA <50 copies/mL by intention to treat analysis. Secondary endpoints were week 24 HIV-1 RNA <50 copies/mL, NVP and RPV pharmacokinetics, safety and fasting lipids, Framingham risk scores (FRS) and Adult Treatment Panel III (ATP-III) lipid goals.

**Results:**

Of 189 eligible patients, we included 50 RPV switchers and 139 NVP controls. Week 12 HIV-RNA was <50 copies/mL in 46/50 switchers (92.0%) which was not different from the hypothesized 90% week 12 suppression rate (p=.431). Forty-four of 50 switchers had week 24 HIV-1 RNA <50 copies/mL compared to 126/139 controls (difference: 2.6%, 95% CI −7.6% to 12.8%, p=.593). NVP plasma concentrations were below detection level in all at week 3. Mean week 1 RPV trough concentration was 0.083 mg/L and comparable to phase III trial data (p=0.747). Adverse events occurred in 36 switchers, the majority (82.0%) were grade one. Two switchers discontinued RPV for side effects. Significant changes over 24 weeks (p<0.001) were observed in switchers on total cholesterol (TC, −0.67 mmol/L, 95% CI −0.50 to 0.83), low density lipoprotein (LDL)-C (−0.36, 95% CI −0.21 to −0.51) and high density lipoprotein (HDL)-C (−0.28, 95% CI −0.20 to −0.35). The TC/HDL-C ratio increased 0.20 (95% CI 0.02 to 0.37; p=.029) and systolic blood pressure decreased 6.0 mmHg (95% CI −1.7 to −10.3; p=.007). The median FRS did not change over 24 weeks (8.4% vs. 7.7%; p=.119). More patients achieved LDL-C (+15%; p=.016) and TC (+25%; p<0.001) ATP-III treatment goals at week 24 on RPV.

**Conclusions:**

A NVP to RPV switch does not influence RPV exposure and results in adequate ongoing HIV-1 suppression. RPV could be an option for patients at risk for cardiovascular diseases.

